# Valaciclovir for Epstein-Barr Virus Suppression in Moderate-to-Severe COPD: A Randomized Double-Blind Placebo-Controlled Trial

**DOI:** 10.1016/j.chest.2023.03.040

**Published:** 2023-04-01

**Authors:** Dermot A. Linden, Hong Guo-Parke, Michael C. McKelvey, Gisli G. Einarsson, Andrew J. Lee, Derek J. Fairley, Vanessa Brown, Gavin Lundy, Christina Campbell, Danielle Logan, Margaret McFarland, Dave Singh, Daniel F. McAuley, Clifford C. Taggart, Joseph C. Kidney

**Affiliations:** a Mater Hospital Belfast, Belfast Health and Social Care Trus, Belfast, Northern Ireland; b Wellcome-Wolfson Institute for Experimental Medicine, School of Medicine, Dentistry & Biomedical Sciences, Belfast, Northern Ireland; c Halo Research Group, School of Pharmacy, Queen’s University Belfast, Belfast, Northern Ireland; d Regional Virus Laboratory, Belfast Health and Social Care Trust, Belfast, Northern Ireland; e Northern Ireland Clinical Trials Unit, Belfast, Northern Ireland; f Royal Victoria Hospital, Belfast, Northern Ireland; g Division of Infection and Immunity, University of Manchester, Manchester, England

**Keywords:** COPD, Epstein-Barr virus, placebo-controlled trial, randomized control trial, valaciclovir

## Abstract

**Background:**

Epstein-Barr virus (EBV) frequently is measured at high levels in COPD using sputum quantitative polymerase chain reaction, whereas airway immunohistochemistry analysis has shown EBV detection to be common in severe disease.

**Research Question:**

Is valaciclovir safe and effective for EBV suppression in COPD?

**Study Design and Methods:**

The Epstein-Barr Virus Suppression in COPD (EViSCO) trial was a randomized double-blind placebo-controlled trial conducted at the Mater Hospital Belfast, Northern Ireland. Eligible patients had stable moderate-to-severe COPD and sputum EBV (measured using quantitative polymerase chain reaction) and were assigned randomly (1:1) to valaciclovir (1 g tid) or matching placebo for 8 weeks. The primary efficacy outcome was sputum EBV suppression (defined as ≥ 90% sputum viral load reduction) at week 8. The primary safety outcome was the incidence of serious adverse reactions. Secondary outcome measures were FEV_1_ and drug tolerability. Exploratory outcomes included changes in quality of life, sputum cell counts, and cytokines.

**Results:**

From November 2, 2018, through March 12, 2020, 84 patients were assigned randomly (n = 43 to valaciclovir). Eighty-one patients completed trial follow-up and were included in the intention-to-treat analysis of the primary outcome. A greater number of participants in the valaciclovir group achieved EBV suppression (n = 36 [87.8%] vs n = 17 [42.5%]; *P* < .001). Valaciclovir was associated with a significant reduction in sputum EBV titer compared with placebo (–90,404 copies/mL [interquartile range, –298,000 to –15,200 copies/mL] vs –3,940 copies/mL [interquartile range, –114,400 to 50,150 copies/mL]; *P* = .002). A statistically nonsignificant 24-mL numerical FEV_1_ increase was shown in the valaciclovir group (difference, –44 mL [95% CI, –150 to 62 mL]; *P* = .41). However, a reduction in sputum white cell count was noted in the valaciclovir group compared with the placebo group (difference, 2.89 [95% CI, 1.5 × 10^6^-7.4 × 10^6^]; *P* = .003).

**Interpretation:**

Valaciclovir is safe and effective for EBV suppression in COPD and may attenuate the sputum inflammatory cell infiltrate. The findings from the current study provide support for a larger trial to evaluate long-term clinical outcomes.

**Trial Registry:**

ClinicalTrials.gov; No.: NCT03699904; URL: www.clinicaltrials.gov

## Graphical Abstract

**Figure:**
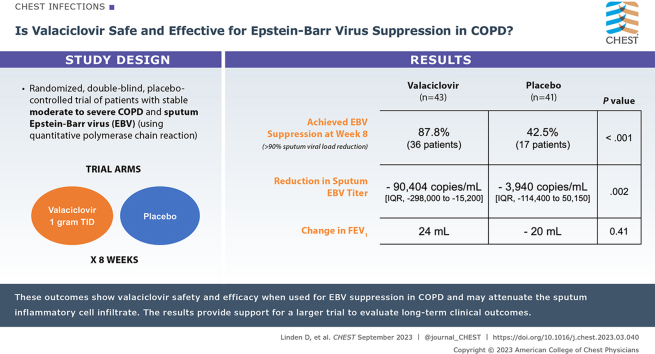

FOR EDITORIAL COMMENT, SEE PAGE 564

**Take-home Points**

**Study Question:** Is valaciclovir safe and effective for Epstein-Barr virus (EBV) suppression in COPD?

**Results:** This study demonstrated that EBV infection, as indicated by shedding of the virus in the sputum, can be suppressed in patients with COPD using valaciclovir (1 g tid for 8 weeks); however, this was not associated with significant improvements in FEV_1_ or quality of life, despite a reduction in sputum total cell count.

**Interpretation:** Valaciclovir is safe and effective for EBV suppression in COPD and may attenuate the sputum inflammatory cell infiltrate. The findings from the current study provide support for a larger trial to evaluate long-term clinical outcomes.

COPD is increasingly recognized as a heterogenous condition.^1^ However, numerous cluster analyses have not led to a consensus regarding disease subtype definitions.^2^, ^3^, ^4^ Furthermore, targeted therapeutic strategies remain limited because of ongoing challenges in characterizing distinct inflammatory endotypes.^5^ Increased numbers of activated neutrophils and macrophages are present in the sputum of patients with COPD.^6^^,^^7^ Also, dysregulation of the adaptive immune response^8^ and increased sputum expression of potent monocyte and CD8^+^ T-cell chemoattractants such as CXCL9, MCP-1, and IP-10 have been shown previously.^9^^,^^10^ A significant correlation exists between disease severity and CD8^+^ T-cell infiltration of the airway epithelium.^11^ This aberrant adaptive immune response culminates in tertiary lymphoid organ (TLO) formation in severe disease.^12^^,^^13^ CD8^+^ T lymphocytes mediate antiviral effector functions and normally undergo apoptosis after viral eradication. Consequently, their infiltration of the airway epithelium in COPD previously generated the hypothesis that chronic viral infection may be implicated in disease pathogenesis.^14^^,^^15^ McManus et al^16^ reported high levels of sputum Epstein-Barr virus (EBV) during COPD exacerbations with similar viral titers in most patients months later, suggesting persistence of infection. A cohort of patients showing negative sputum quantitative polymerase chain reaction (qPCR) results during exacerbation were subsequently shown to have high sputum EBV viral load during stable disease.^16^ This may indicate cyclical virus shedding. In a separate study using immunohistochemistry, the number of airways showing positive staining for latent EBV antigens was increased significantly in severe COPD.^17^ In small airways, EBV antigen detection was associated with secretory IgA deficiency and CD8^+^ lymphocyte accumulation.^17^ More recently, single-cell RNA sequencing showed that dendritic type 2 cells expressing *EBI2* play a key mechanistic role in TLO formation.^13^ We hypothesized that this γ-herpes virus could represent a novel therapeutic target that could be modulated using an established oral thymidine kinase inhibitor. No previous prospective studies have examined herpes virus suppression in COPD. Several randomized trials showed that acyclovir inhibits oropharyngeal EBV shedding in infectious mononucleosis without associated improvement in clinical outcomes.^18^^,^^19^ Notably, EBV suppression with valaciclovir has been shown to improve clinical outcomes in infectious mononucleosis.^20^ Furthermore, Walling et al^21^ found that valaciclovir 1 g tid for 8 weeks inhibited EBV replication with clinical resolution of oral hairy leukoplakia. Valaciclovir is a prodrug of acyclovir with three-fold to five-fold higher bioavailability and may reach levels similar to those of IV acyclovir.^22^ On the basis of these pharmacokinetic data and the precedent of the clinical treatment response demonstrated by Walling et al, we elected to study valaciclovir using a dose of 1 g tid for 8 weeks. The aim of this study was to evaluate the safety, efficacy, and clinical effects of valaciclovir for EBV suppression in COPD.

## Study Design and Methods

### Study Design and Patients

This was a randomized double-blind placebo-controlled allocation-concealed clinical trial conducted at Mater Hospital Belfast, Northern Ireland. Trial management, statistical support, and data monitoring and management was facilitated by the Northern Ireland Clinical Trials Unit (NICTU). The trial was sponsored by Belfast Health and Social Care Trust and received approval from the Office of Research Ethics Committees Northern Ireland (Identifier: 18/NI/0106). Clinical trial authorization was granted by Medicines and Healthcare Products Regulatory Agency. The study safety data were monitored every 6 months by an independent data monitoring and ethics committee. The trial was registered prospectively on EudraCT 2017-004686-28 and ClinicalTrials.gov (Identifier: NCT03699904). The trial protocol and statistical analysis plan, completed before data analysis, are available online from NICTU. Patients who were older than 18 years and had a diagnosis of COPD according to the Global Initiative for Chronic Obstructive Lung Disease criteria (FEV_1_ to FVC ratio of < 0.7 after bronchodilator administration) were screened for trial participation. The main inclusion criteria were sputum EBV detection (evaluated by qPCR) and moderate or severe airflow limitation (FEV_1_ of 30%-80% predicted). Patients were excluded if they had experienced a recent exacerbation (in the previous month) or had a diagnosis of asthma, bronchiectasis (CT scan proven), or interstitial lung disease. Patients continued maintenance COPD therapy. The inclusion and exclusion criteria are detailed in the trial protocol and are available online from NICTU. The trial was conducted in accordance with good clinical practice guidelines and the ethical principles described in the Declaration of Helsinki. All patients provided written informed consent before study entry.

### Randomization and Masking

Eligible participants were enrolled by the study investigators and assigned randomly (1:1) to valaciclovir 1 g tid or matching placebo for 8 weeks according to a prespecified randomization schedule. Mixed block sizes and no stratification were used. The randomization schedule was generated by an independent NICTU statistician using nQuery Advisor (Statsols). Both participants and investigators were masked to group assignment. Masking was achieved by gelatin encapsulation. Valaciclovir capsules contained valaciclovir tablets surrounded by microcrystalline cellulose, whereas placebo capsules contained microcrystalline cellulose only. Both valaciclovir and placebo capsules were identical.

### Procedures

The trial involved a screening visit, a baseline visit (visit 1), and 8 weeks of double-blind treatment with valaciclovir (1 g tid for 8 weeks) or matching placebo with scheduled follow-up visits at week 4 (visit 2) and week 8 (visit 3). Participants continued with their allocated treatment schedule until they attended visit 3. A final follow-up phone call occurred at week 12 to assess adverse events (AEs) and exacerbations. The demographic and clinical details of each participant were recorded at the baseline visit. All study visits included detailed clinical assessment including documentation of medical history, medications, assessment of exacerbations, and sputum collection for quantification of EBV and exploratory outcomes. In accordance with Global Initiative for Chronic Obstructive Lung Disease recommendations, an exacerbation of COPD was defined as “an acute worsening of respiratory symptoms that results in additional therapy.”^23^ Participants underwent measurement of lung function at baseline and week 8 according to American Thoracic Society/European Respiratory Society guidelines.^24^ Quality-of-life questionnaires (COPD Assessment Test and EQ-5D-5L questionnaire) were completed at baseline, week 4, and week 8. Blood samples were collected at baseline and week 8 to assess full blood count, C-reactive protein levels, and serum cytokine levels. Treatment-emergent AEs were assessed clinically for severity, organ system(s) affected, and relatedness. The AE reporting period for this trial began on enrollment and ended 28 days after the completion of study drug administration. All AEs assessed as possibly, probably, or definitely related to the study drug were defined as an adverse reaction. In March 2020, the need to attend hospital during the initial COVID-19 pandemic national lockdown presented an unacceptable risk to patient safety necessitating a substantial amendment to the study protocol. Consequently, the final 12 enrolled patients did not undergo measurement of lung function or biological sample collection for exploratory outcomes.

### Outcomes

The primary efficacy outcome was the suppression of EBV in the sputum measured using qPCR between baseline and week 8. EBV suppression was defined as a 90% reduction in the viral load at week 8. The primary safety outcome was the incidence of serious adverse reactions. Prespecified secondary outcomes included the change in FEV_1_ from baseline to week 8 and drug compliance. Prespecified exploratory outcomes included COPD Assessment Test score, EQ-5D-5L questionnaire score, and changes in sputum cell counts and cytokine levels from baseline to week 8.

### Sputum EBV Quantification and Biomarker Analysis

Nucleic acid was extracted from sputum specimens according to Regional Virus Laboratory Standard Operating Procedures. Full details of the sputum EBV quantification and sputum processing methods are provided in e-Appendix 1.

### Sample Size

Using a χ^2^ test, a sample size of 31 patients per group was calculated to have 90% power at a two-tailed significance level of 0.05 to detect a difference in the primary efficacy outcome of EBV suppression of 70% in the treated group to 30% in the control group. Based on similar studies of patients with COPD, we anticipated an approximately 30% dropout rate. Therefore, the study required the recruitment total of 44 patients per group and an overall total of 88 patients.

### Statistical Analysis

The primary efficacy outcome was analyzed on an intention-to-treat basis using a χ^2^ test, followed by logistic regression adjusting for baseline FEV_1_ as % predicted. A secondary analysis was undertaken on the subset of patients who had an overall drug adherence of at least 70% (per protocol). ORs and 95% CIs were reported for the adjusted analyses. The planned analysis for the primary safety outcome was to compare the two groups using Fisher exact test and to present the relative risk and 95% CIs. A priori-defined subgroup analysis of the primary outcome was performed based on compliance and the ORs and 99% CIs from the treatment × subgroup interaction model were reported. Predefined exploratory subgroup analysis based on EBV suppression (yes or no) also was performed for the lung function outcomes, and analysis of covariance assessed any differences in treatment effects between the subgroups and were reported using the mean difference and 99% CI. The interaction term in both subgroup analyses was the likelihood ratio test.

Continuous outcomes were reported using mean ± SD, or median (interquartile range [IQR]) if appropriate, and treatment groups were compared using independent samples *t* tests (mean and 95% CIs) or nonparametric equivalents. Categorical outcomes were reported using frequencies and percentages, and treatment groups were compared using the χ^2^ test. A paired samples *t* test was used to compare within-group differences at baseline and week 8, and differences were reported as mean and 95% CI. Analyses were on an intention-to-treat basis, and all statistical tests were at the two-sided *P* value of .05. AEs were reported according to number of events and number of patients by treatment group and were presented as risk ratio and 95% CI. The statistical analysis was performed using STATA/IC version 15.1 software (StataCorp LLC). Clinical trial outcomes analyses were performed by the study statisticians according to the predefined statistical analysis plan available online from NICTU. Exploratory data were analyzed using GraphPad Prism (GraphPad). A *P* value of < .05 was considered significant.

## Results

**Figure 1:**
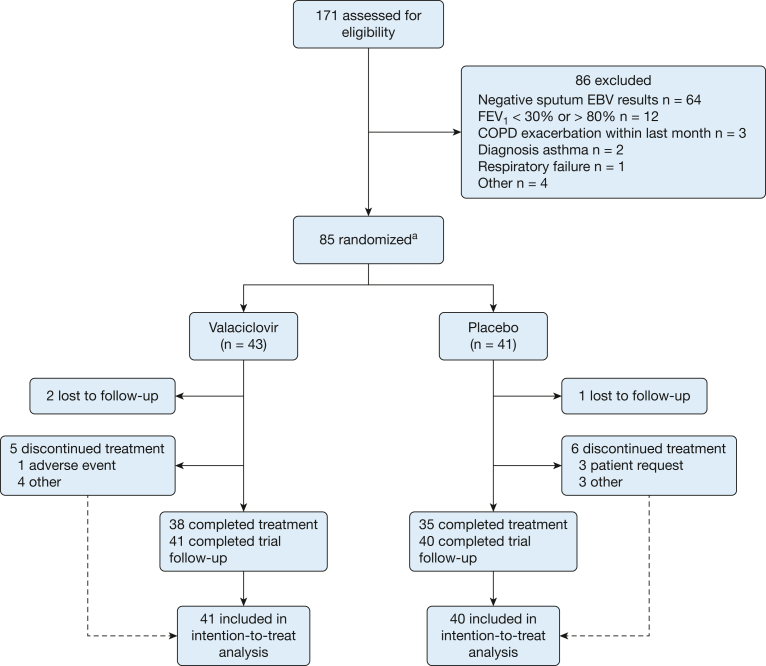
Consolidated Standards of Reporting Trials flow diagram showing the Epstein-Barr Virus Suppression in COPD Study: a randomized double-blind placebo-controlled trial. ^a^n = 85 because one patient withdrew on the same day of randomization, but this was before treatment allocation because the pharmacy still needed to assign a kit code, and so this patient is not included in the analysis. EBV = Epstein-Barr virus.

**Figure 2:**
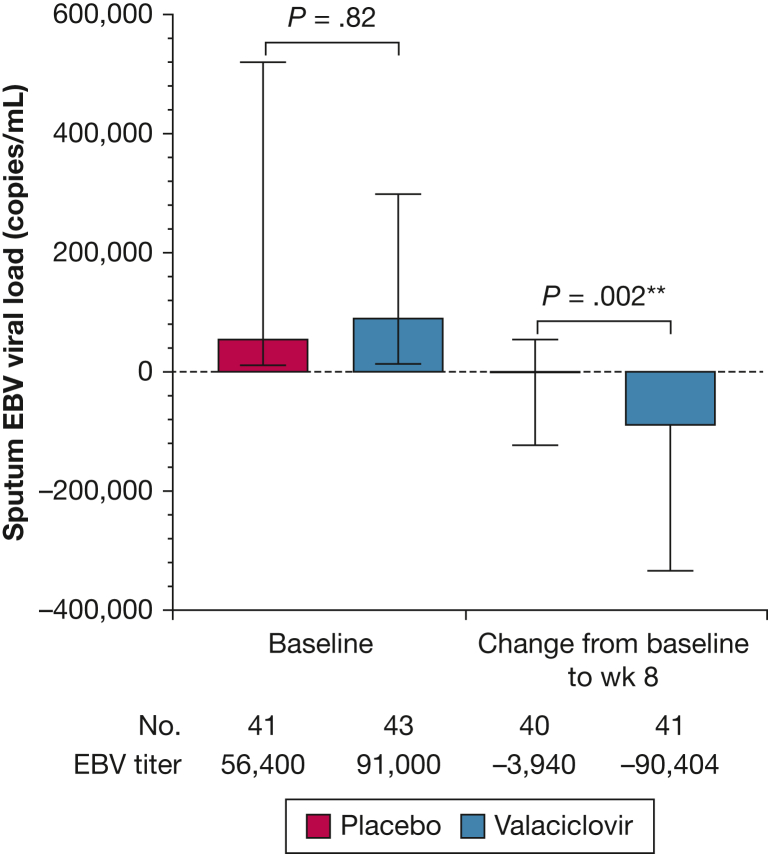
Boxplot showing baseline sputum EBV viral load and change from baseline to week 8. Error bars show the interquartile range. EBV = Epstein-Barr virus.

Between October 2018 and March 2020, 171 patients underwent trial screening. In total, 84 patients were assigned randomly to receive either valaciclovir (n = 43) or placebo (n = 41) (Fig 1). Absence of sputum EBV detection (n = 64) and FEV_1_ outside the defined criteria (n = 12) were the most common reasons for exclusion. Eighty-one patients (96%) completed all trial visits and follow-up and were included in the intention-to-treat analysis of the primary efficacy outcome (n = 41 [95%] in the valaciclovir group and n = 40 [97%] in the placebo group). Three patients were lost to follow-up and were excluded from the analysis. Overall, the baseline demographics and clinical characteristics were similar across both groups, with no major imbalances in prescribed inhaled therapy or number of exacerbations in the previous year (Table 1). Most participants were male (55 [65.5%]), with a mean ± SD age of 61.7 ± 9.0 years. The mean ± SD baseline FEV_1_ was 57.3 ± 13.0% predicted for the valaciclovir group and 56.7 ± 14.8% predicted for the placebo group. The Epstein-Barr Virus Suppression in COPD trial was terminated early because of the initial COVID-19 pandemic national lockdown in the United Kingdom (85 patients were randomized from a planned sample size of 88). The number of participants achieving sputum EBV suppression at week 8 (primary efficacy outcome) was significantly higher in the valaciclovir group than in the placebo group (n = 36 [87.8%] vs n = 17 [42.5%]; *P* < .001) (Table 2). Valaciclovir was associated with a statistically significant reduction in sputum EBV qPCR titer at week 8 compared with placebo (–90,404 copies/mL [IQR, –298,000 to –15,200 copies/mL] vs –3,940 copies/mL [IQR, –114,400 to 50,150 copies/mL]; *P* = .002) (Fig 2). In the valaciclovir group, this corresponded to a reduction in the sputum EBV load to 0 copies/mL [IQR, 0.0-439 copies/mL], and 30 patients (75%) showed negative results on sputum qPCR analysis (e-Table 1). No serious adverse reactions occurred in either arm of the study.

**Table 1:** Baseline Patient Demographic and Clinical Characteristics

Variable	Valaciclovir (n = 43)	Placebo (n = 41)
Demographics		
Age, y	63.1 ± 8.1	60.3 ± 9.8
Male sex	29 (67.4)	26 (63.4)
Smoking status		
Current	19 (44.2)	24 (58.5)
Former	24 (55.8)	17 (41.5)
Smoking history, pack-y	55.3 ± 36.7	48.3 ± 25.9
BMI, kg/m^2^	27.3 ± 5.3	27.2 ± 6.2
Sputum EBV qPCR titer, copies/mL	91,000 (15,200-298,000)	56,400 (11,500-315,000)
Inhaled medications		
LABA	33 (76.7)	36 (87.8)
LAMA	25 (58.1)	21 (51.2)
ICS	29 (67.4)	34 (82.9)
Lung function		
FEV_1_ after bronchodilator administration		
L	1.60 ± 0.58	1.57 ± 0.62
% predicted	57.3 ± 13.0	56.7 ± 14.8
FVC after bronchodilator administration, L	3.21 ± 0.92	3.13 ± 0.94
FEV_1_ to FVC ratio after bronchodilator administration, %	49.44 ± 9.67	49.88 ± 10.23
Transfer factor, % predicted	58.53 ± 16.30 (n = 36)	62.97 ± 19.28 (n = 32)
Cardiovascular comorbidity^a^	15 (34.9)	14 (34.2)
COPD exacerbation in previous 12 mo		
No. (%)	32 (74.4)	29 (70.7)
Median (IQR)	2.5 (1.0-4.0)	2.0 (1.0-4.0)
Resulting in		
Prescription of corticosteroids	2.0 (1.0-3.0)	2.0 (1.0-3.0)
Prescription of antibiotics	2.5 (1.0-4.0)	2.0 (1.0-3.0)
Hospital admission	0.0 (0.0-0.5)	0.0 (0.0-0.0)

Data are presented as No. (%), mean ± SD, or median (IQR). EBV = Epstein-Barr virus; ICS = inhaled corticosteroid; IQR = interquartile range; LABA = long-acting β-agonist; LAMA = long-acting muscarinic antagonist; qPCR = quantitative polymerase chain reaction.

a Includes patients with at least one cardiovascular comorbidity.

**Table 2:** Primary Efficacy Outcome: Sputum EBV Suppression in the Intention-to-Treat Population (n = 81)

Variable	Valaciclovir (n = 41)	Placebo (n = 40)	OR (95% CI)	*P* Value
Sputum EBV suppression at wk 8				
Intention-to-treat analysis^a^^,^^b^	36 (87.8)	17 (42.5)	...	< .001
Intention-to-treat adjusted analysis^c^^,^^d^	...	...	9.8 (3.2-30.1)	< .001
Per-protocol analysis^a^	31 (88.6), n = 35	15 (44.1), n = 34	...	< .001
Per-protocol adjusted analysis^c^^,^^d^	...	...	9.9 (2.8-34.4)	< .001

Primary efficacy outcome subgroup analysis based on compliance	No.	EBV Suppression	No.	EBV Suppression	OR (95% CI)	*P* Value
≥ 80% compliance	33	30 (90.9)	29	13 (44.8)	12.3 (2.0-76.9)	.92^e^
60%-79% compliance	4	3 (75)	7	3 (42.9)	4.0 (0.1-141.5)	.92^e^
< 60% compliance	4	3 (75)	4	1 (25)	9.0 (0.1-604.0)	.92^e^

Data are presented as No. (%), unless otherwise indicated. EBV = Epstein-Barr virus.

a Primary analysis.

b χ^2^ test.

c Logistic regression.

d Adjusted analysis for FEV_1_ % predicted.

e Interaction term.

Because of the initial COVID-19 national lockdown restrictions in the United Kingdom, the final 12 enrolled patients were unable to undergo lung function measurements at the end of treatment. Seventy patients had lung function measured at baseline and week 8 (Table 3). No statistically significant differences were found in the secondary outcomes. In the valaciclovir group, a 24-mL numerical increase in the FEV_1_ at week 8 was found compared with a 20-mL FEV_1_ reduction in the placebo group (difference, –44 mL [95% CI, –150 to 62 mL]; *P* = .41). The overall study drug compliance was 86.2 ± 14.5% in the valaciclovir group compared with 81.4 ± 23.3% in the placebo group (difference, –4.8% [95% CI, –13.4% to 3.8%]; *P* = .27).

**Table 3:** Change in Lung Function From Baseline to Week 8 in Patients With Measurements at Both Time Points

Variable	Valaciclovir (n = 36)	Placebo (n = 34)	Difference (95% CI)	*P* Value^a^
FEV_1_ after bronchodilator administration, L				
Baseline	1.60 ± 0.61	1.65 ± 0.64	...	...
Wk 8	1.63 ± 0.63	1.63 ± 0.64	...	...
Change from baseline to wk 8	0.024 ± 0.19	–0.020 ± 0.25	–0.044 (–0.15 to 0.062)	.41
FEV_1_ after bronchodilator administration, % predicted				
Baseline	57.5 ± 13.0	58.8 ± 14.8	...	...
Wk 8	58.3 ± 13.8	57.8 ± 14.3	...	...
Change from baseline to wk 8	0.72 ± 7.6	-0.94 ± 8.7	–1.66 (–5.55 to 2.22)	.40
FEV_1_ to FVC after bronchodilator administration, %				
Baseline	49.1 ± 9.8	51.2 ± 9.8	...	...
Wk 8	49.7 ± 9.8	51.4 ± 9.8	...	...
Change from baseline to wk 8	0.58 ± 3.80	0.23 ± 4.37	–0.36 (–2.31 to 1.59)	.72
PEF, L/s				
Baseline	4.6 ± 1.8	4.6 ± 1.6	...	...
Wk 8	4.7 ± 1.7	4.6 ± 1.6	...	...
Change from baseline to week 8	0.08 ± 0.7	0.01 ± 0.7	–0.072 (–0.41 to 0.27)	.67
TLCO, % predicted	n = 29	n = 28	...	...
Baseline	58.2 ± 17.8	64.6 ± 17.5	...	...
Wk 8	59.0 ± 19.47	64.4 ± 16.9	...	...
Change from baseline to wk 8	0.79 ± 7.0	–0.21 ± 6.1	–1.01 (–4.50 to 2.48)	.57

Data are presented as mean ± SD, unless otherwise indicated. Changes are calculated on the basis of available measurements for both time points. PEF = peak expiratory flow; TLCO = transfer factor for carbon monoxide.

a Independent samples *t* test.

**Figure 3:**
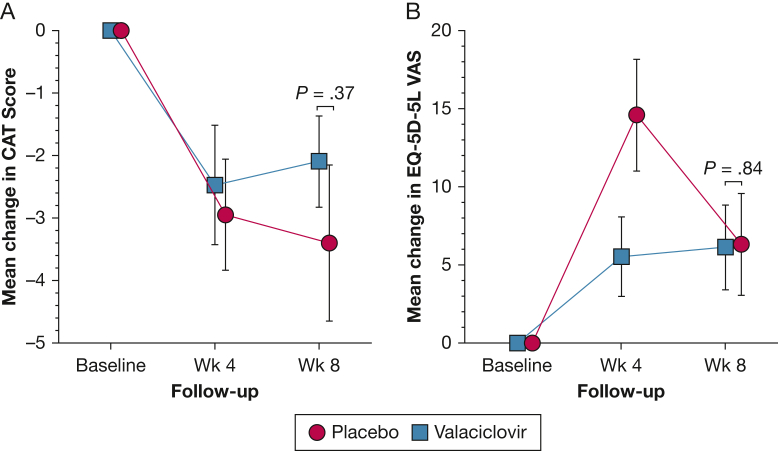
A, B, Graphs showing effect of intervention on symptom scores and quality of life quantified at baseline, week 4, and week 8 using CAT (A) and EQ-5D-5L (B), respectively. CAT = COPD Assessment Test; VAS = visual analogue scale.

Quality-of-life data were available for all 81 patients in the intention-to-treat population (Fig 3). The mean COPD Assessment Test score decreased by 2.1 ± 4.7 in the valaciclovir group compared with a decrease of 3.4 ± 7.9 in the placebo group (difference, –1.3 [95% CI, –4.2 to 1.6]; *P* = .37). In the valaciclovir group, the mean EQ-5D-5L questionnaire utility index increased by 0.02 ± 0.2 compared with an increase of 0.08 ± 0.2 in the placebo group (difference, –0.06 [95% CI, –0.04 to 0.1]; *P* = .23). Additional quality-of-life data can be found in e-Figures 1 and 2 and e-Tables 2 and 3. In an unadjusted analysis, no between-group difference was found in the number of exacerbations (mean difference, –0.34 (95% CI, –0.71 to 0.04; *P* = .08).

**Figure 4:**
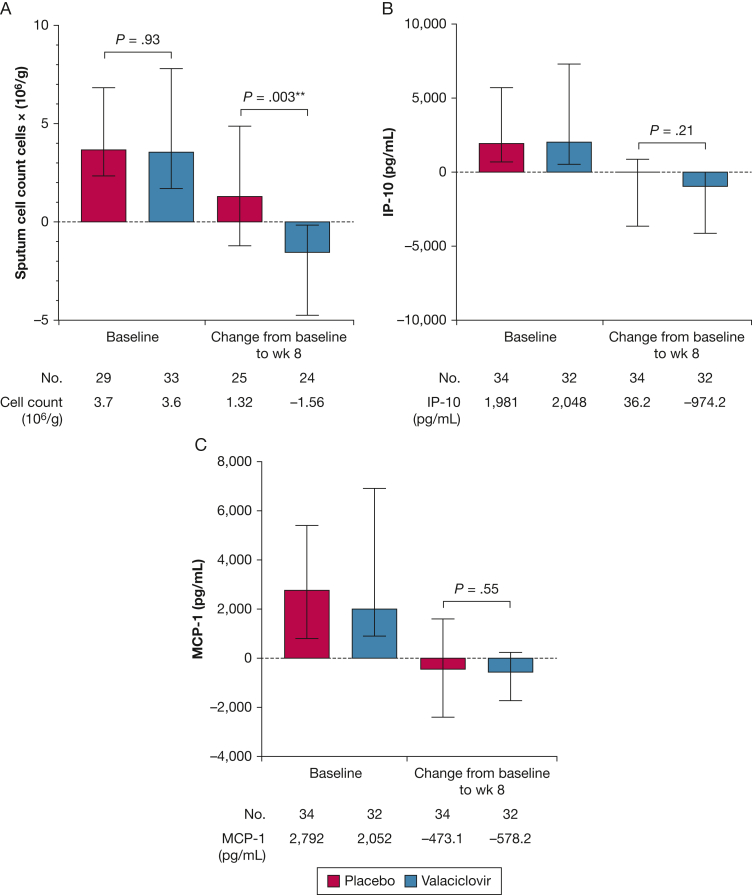
A-C, Boxplots showing baseline sputum biomarkers and mean change from baseline to week 8: sputum total cell count (A), IP-10 (B), and MCP-1 (C) quantified at baseline and at week 8 using trypan blue exclusion method and enzyme-linked immunosorbent assay, respectively. ^a^*P* < .01, Mann-Whitney U test.

The sputum total cell count was reduced significantly at week 8 in the valaciclovir group (difference, 2.9 × 10^6^ [95% CI, 1.5 × 10^6^-7.4 × 10^6^]; *P* = .003) (Fig 4). No significant between-group differences in absolute sputum neutrophil, macrophage, or eosinophil counts were found at week 8. However, neutrophil and macrophage counts were reduced (> 50% reduction) within the valaciclovir group, reaching statistical significance for macrophages (e-Table 4). No treatment effects on sputum cell percentages were found (e-Table 5). Treatment with valaciclovir was associated with a six-fold within-group reduction in the concentration of sputum IP-10 (2,048 pg/mL vs 339.6 pg/mL; *P* = .008) and a 1.6-fold decrease in sputum MCP-1 at week 8 (2,052 pg/mL vs 1,280 pg/mL; *P* = .016). These changes failed to reach between-group significance (Fig 4). No between-group differences were found for sputum IL-6, IL-1β, or ENA-78 (e-Table 6). Treatment with valaciclovir was not associated with any between-group differences in serum C-reactive protein, IL-1β, IL-8, or blood white cell counts (e-Fig 3).

No safety concerns were identified during the conduct of the study, and the incidence of treatment-emergent AEs was comparable in both groups (Table 4). No difference was found in the number of patients affected by AEs in either group (valaciclovir, n = 26 [60.5%] vs placebo, n = 31 [75.6%]; relative risk, 0.80 [95% CI, 0.59-1.08]; *P* = .17).

**Table 4:** Treatment-Emergent AEs in the Safety Population (n = 84)

Variable	Valaciclovir (n = 43)	Placebo (n = 41)	RR (95% CI)	*P* Value
Any AE	50 (50.0)	50 (50.0)	...	...
Patients reporting AE(s)	26 (60.5)	31 (75.6)	0.80 (0.59-1.08)	.17
Adverse reactions	13 (61.9)	8 (38.1)	1.31 (0.59-2.93)	.61
Patients reporting adverse reaction(s)	11 (25.6)	8 (19.5)	...	.49
Unexpected adverse reactions	2 (100.0)	0 (0.0)	...	.49
Serious AEs	0 (0.0)	1 (100.0)	...	.49
Serious adverse reactions	0 (0.0)	0 (0.0)	...	...
Most frequent AEs^a^				
GI disorders	11 (22.0)	10 (20.0)	0.95 (0.44-2.05)	1.00
Respiratory, thoracic, and mediastinal disorders	5 (10.0)	10 (20.0)	0.48 (0.16-1.46)	.22
Hematologic or biochemical blood abnormality	9 (18.0)	6 (12.0)	1.33 (0.46-3.87)	.76

Data are presented as No. (%), unless otherwise indicated. A patient reporting more than one event in a category is counted only once. AE = adverse event; RR = relative risk.

a Those events with an incidence of ≥ 10% in either group.

We conducted a post hoc analysis to examine the effect of sputum EBV suppression, regardless of treatment allocation, on change in FEV_1_ at week 8 (e-Table 7). In the EBV suppression group, a 26-mL numerical increase in FEV_1_ was found compared with a 54-mL reduction in the EBV persistence group (difference, –80 mL (95% CI, –190 to 26 mL). A further post hoc analysis of change in FEV_1_ regardless of treatment allocation and excluding patients who experienced an exacerbation during treatment (e-Table 8) found those with sputum EBV suppression who did not experience an acute COPD exacerbation showed a 44-mL increase in the FEV_1_ compared with a 34-mL reduction in FEV_1_ in patients with persistence of EBV at week 8 (difference, –78 mL; 95% CI, –200 to 42 mL). The pre-specified analysis of covariance did not demonstrate any statistically significant changes in lung function.

## Discussion

In this randomized double-blind placebo-controlled trial of patients with moderate to severe COPD, valaciclovir was shown to be safe and effective for the suppression of EBV. We used valaciclovir at a dose of 1 g tid on the basis of a previous open-label study of oral hairy leukoplakia by Walling et al.^21^ Using this dose, 36 of 41 patients in the intervention group showed EBV suppression at week 8 and 30 showed negative sputum qPCR results. Spontaneous EBV suppression was found in the placebo group at a higher rate than previously reported.^16^ However, 30 patients (75%) in the placebo group still showed detectable EBV in the sputum at week 8. This finding may indicate that, akin to other human herpes viruses, EBV is capable of a cyclical pattern of virus shedding in the lower airway in COPD. Importantly, the study intervention was well tolerated with no serious adverse reactions and a modest number of AEs in each group. The compliance data suggest that this treatment is acceptable to the wider COPD population.

This study was not designed to have adequate power to detect changes in lung function. However, we found a modest 24-mL numerical FEV_1_ increase in the valaciclovir group, resulting in a 44-mL overall FEV_1_ between-group difference that failed to reach statistical significance. This is lower than the previously described minimum clinically important difference.^25^ Importantly, most participants were prescribed inhaled triple therapy, which may have diminished the potential to delineate an FEV_1_ change. A further unfortunate limitation of the study is that the final 12 patients enrolled had missing FEV_1_ data at week 8 because of mandatory COVID-19 restrictions, which further reduced the potential to observe significant treatment effects on lung function. It should be noted that the antiinflammatory drugs currently used in clinical practice for the treatment of COPD, namely inhaled corticosteroids and the phosphodiesterase type 4 inhibitor roflumilast, cause relatively small improvements in FEV_1_ (approximately ≤ 70 mL), which have been demonstrated using larger sample sizes and a longer duration of treatment than in the current study.^26^^,^^27^ We found no difference in self-reported quality of life, suggesting that treatment of EBV does not ameliorate the chronic respiratory symptom burden in the short term.

Our finding that valaciclovir was associated with a reduction in the sputum total cell count may suggest attenuation of the sputum inflammatory cell infiltrate. The absolute neutrophil and macrophage counts were reduced by valaciclovir treatment (by > 50%), contributing to the significant treatment effect on the total cell count. One might hypothesize that persistent EBV shedding contributes to chronic airway immune activation and immune cell recruitment. However, it must be acknowledged that the current study did not demonstrate a definitive between-group difference in sputum cytokine concentrations, and any potential immunomodulatory mechanism of valaciclovir remains to elucidated. The six-fold within-group reduction in IP-10 may warrant further examination. Macrophages release IP-10, which is chemotactic for CD4^+^ and CD8^+^ T lymphocytes (via CXC3 receptor).^28^ Macrophage function often is dysregulated in COPD.^29^^,^^30^ IP-10 is induced by interferon-γ through the Th1-mediated immune response and has both antiviral and antibacterial activity.^31^^,^^32^ IP-10 also is expressed strongly in TLOs, which are associated with COPD progression.^13^^,^^33^ A study by Naessens et al^13^ found that dendritic type 2 cells are the most potent inducers of follicular helper T cells in COPD. These dendritic type 2 cells show a unique migratory signature including *EBI2* expression, known to control immune cell organization during TLO formation.^13^ *EBI2* expression is upregulated strongly in response to EBV infection,^34^ suggesting that the virus may influence the complex milieu of chronic adaptive immune stimulation in COPD during tertiary lymphoid tissue formation.

Our study has some limitations. It is important to consider the spectrum of antiviral activity of valaciclovir. Polosukhin et al^17^ previously reported cytomegalovirus in 12.9% to 34.4% of small airways in COPD, and although valaciclovir has moderate efficacy against cytomegalovirus, it currently is licensed for prophylaxis only when valganciclovir or ganciclovir cannot be used. Valaciclovir has more potent efficacy against herpes simplex virus; however, this is detectable in only 13% of patients with stable COPD,^35^ and subsequently the potential for a collateral treatment effect on other concurrent herpes viruses is likely to have been modest. We acknowledge that our study was not adequately powered for clinical efficacy, and thus the real-life benefit of this antiviral treatment remains to be elucidated. The relatively small size of the trial also meant that despite randomization, unforeseen imbalances could have arisen. An imbalance was present in baseline inhaled corticosteroids use, and although this was not statistically significant, a possibility remains that this could have contributed to a confounding effect. In the context of important baseline variables, we did not collect data prospectively on vaccination status for influenza or herpes zoster. Finally, it should be acknowledged that qPCR cannot discriminate between latent and active viral replication. Theoretically, it is possible that the high levels of detected EBV DNA could be latent. However, in our view, the more likely explanation is that most represented actively replicating virus. Alternative diagnostic methods such as viral culture have been replaced by qPCR, which is now the gold standard methodology, and we believe this justified its use in our study because quantitation was central to the measured outcome.

Beyond COPD, EBV and other herpes viruses have been targeted therapeutically in idiopathic pulmonary fibrosis. Blackwell et al^36^ recently reported safety and tolerability of valganciclovir as an add-on therapy. The study was of patients with circulating EBV antibodies and did not quantify EBV viral load using qPCR. To our knowledge, this is the first study to suppress viral load in pulmonary disease. We recruited from a heterogeneous population with moderate to severe COPD. However, 73% of these patients had experienced an exacerbation in the previous year, with a median number of two exacerbations. Novel adjunctive therapies such as azithromycin and roflumilast recently expanded the treatment arsenal against exacerbations.^27^ However, despite their proven clinical efficacy, these medications often incur significant side effects, including risk of nontuberculous mycobacterial infection, antibiotic resistance, cardiac arrhythmia, and GI symptoms, which may limit their use in the long term. Valaciclovir could represent an attractive adjunctive therapy by virtue of its favorable side-effect profile; however, this study was not large or long enough to study treatment effects on exacerbations. Nevertheless, the positive primary end point findings regarding EBV suppression, coupled with the observed treatment effect on sputum total cell counts, provide support to perform a longer and larger multicenter study focused on the impact of EBV suppression on clinical outcomes in COPD.

## Interpretation

Valaciclovir is safe and effective for EBV suppression in COPD and may attenuate the sputum inflammatory cell infiltrate. The findings from the current study provide support for a larger trial to evaluate long-term clinical outcomes.

## Funding/Support

This study was funded by the Mater Hospital YP Trustee Fund (registered with The Charity Commission for Northern Ireland [Identifier: NIC100084]). D. A. L. has received salary and research costs from Mater Hospital YP Trustees. M. C. M. reports funding from Medical Research Council (MRC) for current post [Grant MR/T016760/1] and previous doctoral study [Grant MR/P022847/1]. D. F. M. reports institutional grants from the UK National Institute for Health and Care Research (NIHR) and the Wellcome Trust. C. C. T. has received grants from MRC, NIHR, NICHS and Chiesi Farmaceutici. J. C. K. reports grants from Chest Heart and Stroke NI, NIHR, and Mater Hospital YP Trustees.

## Financial/Nonfinancial Disclosures

The authors have reported to *CHEST* the following: D. A. L. has received speaker fees from GlaxoSmithKline. H. G.-P. has received grants from Pfizer. M. C. M reports funding from Medical Research Council (MRC). G. G. E. and A. J. L. report funding from IMI. D. S. has received fees for consultancy from Aerogen, AstraZeneca, Boehringer, Chiesi, Cipla, CSL Behring, Epiendo, Genetech, GlaxoSmithKline, Glenmark, Gossamerbio, Kinaset, Menarini, Novartis, Pulmatrix, Sanofi, Synairgen, Teva, and Theravance. D. F. M. reports funding from GlaxoSmithKline and personal fees for consultancy from GlaxoSmithKline, SOBI, Peptinnovate, Boehringer Ingelheim, and Bayer. C. C. T. reports grants from MRC, NIHR, NICHS and Chiesi. J. C. K. reports grants from Pfizer UK. None declared (D. J. F., V. B., G. L., M. M., C. C., D. L.).

## Supplementary Data

e-Online Data
