# Supplementary material for: Valaciclovir for Epstein-Barr Virus Suppression in Moderate-to-Severe COPD: A Randomized Double-Blind Placebo-Controlled Trial
Source: Chest. 2023 Apr 1;164(3):625–36. doi: 10.1016/j.chest.2023.03.040 (PMC10808072; doi:10.1016/j.chest.2023.03.040)
Supplement: e-Online Data [file mmc1.docx]

**Valaciclovir for Epstein-Barr virus suppression in moderate-to-severe COPD (EViSCO): A randomised, double-blind, placebo-controlled trial.**

**Supplementary material**

**Contents:**

| Data Monitoring and Ethics Committee members | 3 |
| --- | --- |
| Supplementary Methods | 4 |
| Sputum processing methods | 4 |
| Sputum EBV quantification | 4 |
| EBV Quantitative PCR analysis and viral load determination | 4 |
| Sputum supernatant cytokine measurements | 5 |
| Blood sample collection, processing and ELISA measurements | 5 |
| Supplementary Figures | 6 |
| Supplementary Figure E1. COPD Assessment Test score by visit | 6 |
| Supplementary Figure E2. EQ-5D-5L VAS score by visit | 6 |
| Supplementary Figure E3: Effect of intervention on changes in serum CRP, serum cytokines and peripheral white blood cell counts by treatment group. | 7 |
| Supplementary Tables | 8 |
| Supplementary Table E1. Sputum EBV descriptive statistics at baseline, week 4 and week 8. | 8 |
| Supplementary Table E2. COPD Assessment test symptom domain scores by visit | 8 |
| Supplementary Table E3. EQ-5D-5L symptom domains, VAS and index scores by visit | 9 |
| Supplementary Table E4. Sputum cell counts from baseline to week 8 | 9 |
| Supplementary Table E5. Sputum cell count percentage (%) from baseline to week 8. | 10 |
| Supplementary Table E6. Change in the sputum cytokine levels in the valaciclovir and placebo groups from baseline to week 8 | 10 |
| Supplementary Table E7. Post-hoc analysis of change in lung function and quality of life based upon EBV suppression at week 8 irrespective of treatment allocation. | 11 |
| Supplementary Table E8. Post-hoc analysis of change in lung function from baseline to week 8 based upon EBV suppression irrespective of treatment allocation | 12 |

**Data Monitoring and Ethics Committee members:**

Professor Lorcan McGarvey (Chair), Queen’s University Belfast, UK

Dr Thelma Craig, Mater Hospital Belfast, UK

Dr Matyas Szigeti, Imperial College London, UK

**Supplementary Methods**

**Sputum processing methods**

Sputum samples were collected via spontaneous production or if necessary sputum induction. Sputum was collected on ice and processed immediately at 4^o^C. The expectorated sputum was aliquoted into two specimen containers at each study visit. One sample was immediately transferred to the Regional Virus Laboratory for primary efficacy outcome analysis (EBV quantitative PCR analysis) whilst the remaining sample was transferred for exploratory outcome measurements. Sputum plugs were selected out of the sample using curved forceps, from saliva and transferred onto a petri dish lid. The sputum plugs were gathered into one mass using blunt ended forceps and condensed by moving the entire mass around the lid with small circular motions with the aim of spreading saliva across the lid but to keep sputum in one mass. A volume of 1 X Dulbecco’s PBS (DPBS) equal to 8 times the weight (W) of the selected sputum plug was added to the sputum. The sputum sample was dispersed by repeated gentle aspiration using a plastic pipette and vortexed for 15 seconds. The resulting sample solution was placed on a bench rocker with ice for 15 minutes and then centrifuged at 790xg for 10 minutes at 4^o^C (brake off). Following centrifugation a volume of sample supernatant equal to 4 times the weight of the sputum plugs (4 x W) was pipetted into a sterile 15ml tube and centrifuged at 1500g for a further 10 minutes at 4^o^C (brake off). The supernatant from this step was aliquoted and stored at -80^o^C for cytokine analysis and measurement of protease activity. The remaining sample was then diluted with a 4-fold volume of 0.1 % sputolysin solution and dispersed with repeated gentle aspiration in a plastic Pasteur pipette and vortexed for 15 seconds. The samples were then incubated for 15 minutes on a bench rocker at room temperature. The sputum suspension was then filtered through 48μm nylon gauze placed in a funnel. The filtrate was kept on ice whilst performing the total cell count. Using the filtrate solution cytospin slides were prepared for differential white cell count. Slides were air dried at room temperature, then fixed with methanol for 10 minutes. Slides were stained using Giemsa staining (Speedy-Diff complete kit, Clin-Tech Ltd). All slides were counted independently by two readers who were blinded to clinical details and treatment allocation. In the event the counts differed by more than 10% the slides were reviewed by a third reader. All sputum white cell differential counts were conducted prior to unblinding of treatment allocation. Differential counts which had <100 leukocytes total or >80% squamous were excluded as unevaluable samples. Due to the initial COVID-19 UK national lockdown restrictions and laboratory closures it was not possible to conduct exploratory sputum processing for the final 12 participants at the end of treatment. The remaining subjects with evaluable slides (*n*=44) were used for analyses.

**Sputum EBV quantification**

**Nucleic acid extraction**

Nucleic acid was extracted from sputum specimens according to Regional Virus Laboratory Standard Operating Procedures. In brief, clinical sputum specimens were homogenised using an equal volume of Sputasol reagent (Thermo Scientific, Manchester, UK). Total viral nucleic acid was then extracted using a MagNA Pure 96 instrument and Viral NA Small Volume Kit (Roche Diagnostics, Burgess Hill, UK) with 200µl input volume. Purified nucleic acid was eluted in a 100µl volume and used for quantitative PCR analysis.

**EBV Quantitative PCR analysis and viral load determination**

Quantitative PCR was undertaken by an experienced BHSCT laboratory technician according to Regional Virus Laboratory Standard Operating Procedures, which are validated for routine diagnostic use in a regional NHS/HSC Reference Laboratory, under UKAS Accreditation / ISO 15189, Laboratory No. 8704. In brief, nucleic acid extracts were tested using the RealStar® EBV PCR Kit 2.0 (Altona Diagnostics, Hamburg, Germany). This assay is CE-IVD approved for in vitro diagnostic use, and quantitative detection of EBV in human clinical specimens. The assay was calibrated for quantitative use with Altona Quantification Standards, which are calibrated against the 1st WHO International Standard for EBV, NIBSC code 09/260.

**Sputum supernatant cytokine measurements**

The ELLA platform (Proteinsimple (Biotechne) San Jose, California) was used to measure sputum supernatant cytokine concentrations. The ELLA cytokine detection system is a high-throughput platform based on four parallel single-plex microfluidics ELISA assays which are run in triplicate within cartridges. A multi-analyte immunoassay (IP-10, MCP-1, IL-6 and ENA-78) was conducted using customised 4-plex cartridges. Sputum IL-1β was quantified using single-plex ELLA cartridges (IL-1β Cat# SPCKB-PS-000216). The assays were conducted in accordance with the manufacturer’s instructions. Sputum supernatant samples were allowed to thaw to room temperature immediately before cytokine quantification. All samples were diluted with the manufacturer’s sample diluent to a final sample volume of 50 μL per well. For the 4-plex immunoassays sputum supernatant samples were diluted using a 2-fold dilution with the sample diluent provided according to the manufacturer’s instructions. A 5-fold dilution was used for IL-1β. Two levels of quality controls (high and low level controls) for each analyte were included in each run to ensure assay performance. All sputum immunoassays were conducted prior to treatment unblinding in a GCLP accredited laboratory.

**Blood sample collection, processing and ELISA measurements**

Peripheral venous blood samples were drawn by the researcher at scheduled study visits for full blood count and serum biomarker measurement. Samples were stored in vacutainer tubes (Greiner Bio-One Ltd, Stonehouse UK). Samples were stored on ice following collection and during transit to the laboratory. On arrival in the laboratory, blood samples were centrifuged at 1500g for 10 minutes in a pre-cooled centrifuge at 4°C. The supernatant resulting from this step was designated serum. Immediately the supernatant component (serum) was aliquoted into a clean polypropylene tube using a Pasteur pipette whilst avoiding disturbing the cell layer at the base of the vacutainer tube. The serum was apportioned into 0.5 ml aliquots and stored at – 80°C. Serum CRP was measured via ELISA (R&D Systems, Abingdon, UK, Cat# DY1707). Serum IL-8 and IL-1β were also measured using the ELLA platform (Proteinsimple (Biotechne) San Jose, California). 50 μL of serum samples was added to ELLA single-plex IL-8 (Cat# SPCKB-PS-000230) and IL-1β (Cat# SPCKB-PS-000216) cartridges. All assays were conducted accordong to the manufacturers SOP. Two levels of quality controls (high and low level controls) for each analyte were included in each run to ensure assay performance. All sputum immunoassays were conducted prior to treatment unblinding in a GCLP accredited laboratory.

**Supplementary Figures**

**Supplementary Figure E1.** **COPD Assessment Test score by visit.**

Error bars show SEM.

**Supplementary Figure E2:** **EQ-5D-5L VAS score by visit**

Error bars show SEM.

**** ****

**** ****

**Supplementary Figure E3:** **Effect of intervention on changes in serum CRP, serum cytokines and peripheral white blood cell counts by treatment group.**

Error bars show interquartile range.

| **Supplementary Table E1. Sputum EBV descriptive statistics at Baseline, week 4 and week 8** | | | | | | | |
| --- | --- | --- | --- | --- | --- | --- | --- |
|  | | **Valaciclovir** | | | **Placebo** | | |
|  |  | **Baseline** | **Week 4** | **Week 8** | **Baseline** | **Week 4** | **Week 8** |
|  |  | **n=43** | **n=34** | **n=41** | **n=41** | **n=37** | **n=40** |
| EBV qPCR Titre(copies/ml)* | | 91000 [15200, 298000] | 0.0 [0, 0] | 0.0 [0.0, 439] | 56400 [11500, 315000] | 772 [0.0, 107000] | 17500 [840, 318500] |
| Sputum EBV detection | Yes | 43 (100.0%) | 3 (8.8%) | 11 (26.8%) | 41 (100.0%) | 22 (59.5%) | 30 (75.0%) |
|  | No | 0 (0.0%) | 31(91.2%) | 30 (73.2%) | 0 (0.0%) | 15 (40.5%) | 10 (25%) |
| Data are median [IQR] | | | | | | | |

| **Supplementary Table E2. COPD Assessment Test symptom domain scores by visit** | | | | | | |
| --- | --- | --- | --- | --- | --- | --- |
|  | **Valaciclovir** | | | **Placebo** | | |
|  | **Baseline**  **n=43** | **Week 4**  **n=40** | **Week 8**  **n=41** | **Baseline**  **n=41** | **Week 4**  **n=37** | **Week 8**  **n=40** |
| Cough | 3.1 (1.3) | 2.7 (1.2) | 2.6 (1.3) | 3.2 (1.3) | 2.6 (1.5) | 2.8 (1.4) |
| Phlegm/ mucus | 3.0 (1.4) | 2.8 (1.4) | 2.9 (1.4) | 3.1 (1.5) | 2.7 (1.3) | 2.5 (1.3) |
| Chest Tightness | 2.1 (1.5) | 1.9 (1.5) | 2.0 (1.5) | 2.4 (1.7) | 1.6 (1.6) | 1.8 (1.7) |
| Breathlessness | 3.8 (1.4) | 3.4 (1.4) | 3.4 (1.6) | 4.2 (1.4) | 3.8 (1.4) | 3.8 (1.6) |
| Limited Activities at Home | 2.7 (1.8) | 2.7 (1.9) | 2.8 (1.8) | 3.4 (1.6) | 3.2 (1.6) | 3.2 (1.6) |
| Confidence in Leaving Home | 2.3 (1.9) | 2.1 (1.8) | 2.3 (1.9) | 2.6 (1.9) | 2.6 (2.0) | 2.5 (2.2) |
| Sleeps Soundly | 2.8 (1.8) | 2.9 (1.6) | 2.7 (1.7) | 3.2 (1.8) | 2.8 (1.6) | 2.5 (1.8) |
| Energy | 3.6 (1.3) | 3.1 (1.3) | 3.0 (1.4) | 3.8 (1.1) | 3.3 (1.3) | 3.4 (1.5) |
| Total | 23.3 (8.7) | 21.4 (8.7) | 21.8 (9.4) | 25.9 (8.2) | 22.6 (8.5) | 22.3 (10.4) |
| Data is presented as the mean (SD) of each CAT questionnaire parameter at baseline, week 4 and week 8 by treatment group. | | | | | | |

| **Supplementary Table E3. EQ-5D-5L symptom domains, VAS and index scores by visit** | | | | | | |
| --- | --- | --- | --- | --- | --- | --- |
| **EQ: 5D**  **(sub scales)** | **Valaciclovir** | | | **Placebo** | | |
|  | **Baseline**  **n=43** | **Week 4**  **n=40** | **Week 8**  **n=41** | **Baseline**  **n=41** | **Week 4**  **n=38** | **Week 8**  **n=40** |
| Mobility | 34/43 (79.1%) | 31/40 (77.5%) | 31/41 (75.6%) | 37/41 (90.2%) | 33/38 (86.8%) | 33/40 (82.5%) |
| Self-care | 22/43 (51.2%) | 22/40 (55.0%) | 23/41 (56.1%) | 29/41 (70.7%) | 24/38 (63.2%) | 27/40 (67.5%) |
| Usual activities | 35/43 (81.4%) | 30/40 (75.0%) | 29/41 (70.7%) | 36/41 (87.8%) | 33/38 (86.8%) | 35/40 (87.5%) |
| Pain/discomfort | 35/43 (81.4%) | 32/40 (80.0%) | 29/41 (70.7%) | 39/41 (95.1%) | 29/38 (76.3%) | 29/40 (72.5%) |
| Anxiety/Depression | 27/43 (62.8%) | 25/40 (62.5%) | 26/41 (63.4%) | 28/41 (68.3%) | 23/38 (60.5%) | 26/40 (65.0%) |
| EQ: 5D (VAS) | 53.9 (20.8) | 59.2 (22.4) | 59.7 (21.2) | 49.2 (22.6) | 65.2 (19.3) | 56.0 (24.0) |
| Index Score | 0.5 (0.3) | 0.5 (0.3) | 0.5 (0.3) | 0.4 (0.3) | 0.4 (0.3) | 0.4 (0.3) |

| **Supplementary Table E4. Sputum cell counts from baseline to week 8** | | | | | |
| --- | --- | --- | --- | --- | --- |
|  |  | | **Baseline** | **Week 8** | **P value** |
| Sputum  Total cell count  (x10^6^/g) | Placebo* | n=25 | 3.7 [2.4, 6.9] | 5.5 [2.8, 8.6] | 0.07 |
|  | Valaciclovir* | n=24 | 3.7 [2.0, 8.1] | 1.6 [1.0, 3.5] | 0.01 |
| Sputum  Neutrophil  Total cell count  (x10^6^/g) | Placebo† | n=21 | 2.9 [1.9, 7.3] | 3.9 [1.5, 6.3] | 0.94 |
|  | Valaciclovir† | n=23 | 2.7 [1.1, 6.5] | 1.1 [0.5, 6.9] | 0.24 |
| Sputum  Macrophages  Total cell count  (x10^6^/g) | Placebo† | n=21 | 0.4 [0.2, 0.9] | 0.2 [0, 0.7] | 0.8 |
|  | Valaciclovir† | n=23 | 0.86 [0.4, 1.5] | 0.4 [0.1, 0.7] | 0.01 |
| Sputum  Eosinophils  Total cell count  (x10^6^/g) | Placebo† | n=21 | 0.07 [0.02, 0.38] | 0.09 [0.02, 0.58] | 0.85 |
|  | Valaciclovir† | n=23 | 0.05 [0.01, 0.12] | 0.05 [0.01, 0.09] | 0.98 |
| Data are median [IQR]. Data from subjects in whom evaluable sputum samples were available are shown. *P value from Wilcoxon signed rank test. †P value from Mann-Whitney U test. | | | | | |

| **Supplementary Table E5. Sputum cell count percentage (%) from baseline to week 8** | | | | | | |
| --- | --- | --- | --- | --- | --- | --- |
|  |  | | **Baseline** | **Week 8** | **Difference**  **(95% CI)** | **P value** |
| Sputum  Neutrophils (%) | Placebo | n=21 | 87 [75, 91.9] | 81.9 [62, 89] | -5.1 [-12.3, 2.2] | 0.18 |
|  | Valaciclovir | n=23 | 76.5 [56, 88] | 82.4 [68, 91.3] | 5.85 [-4.7, 15.8] | 0.31 |
| Sputum  Macrophages (%) | Placebo | n=21 | 10 [5.1, 21.1] | 10.7 [7.5, 29.7] | 0.7 -3.0, 8.2] | 0.36 |
|  | Valaciclovir | n=23 | 21.3 [10.5, 39.8] | 10.9 [6.8, 30.8] | -10.4 [-15, 2.1] | 0.14 |
| Sputum  Eosinophils (%) | Placebo | n=21 | 1.08 [0.06, 3.06] | 0.98 [0.24, 9.96] | -0.10 -0.95, 1.27] | 0.74 |
|  | Valaciclovir | n=23 | 0.25 [0, 2.79] | 0.45 [0, 4.64] | -0.20 -0.03, 1.00] | 0.27 |
| Data are median [IQR]. Data from subjects in whom evaluable sputum samples were available are shown. *P value from Mann-Whitney U test. | | | | | | |

| **Supplementary Table E6. Change in the sputum cytokine levels in the valaciclovir and placebo groups from baseline to week 8** | | | |
| --- | --- | --- | --- |
|  | **Valaciclovir**  **(n=32)** | **Placebo**  **(n=34)** | **P value** |
| Change in sputum cytokine concentration from baseline to week 8; |  |  |  |
| IP-10 (pg/ml) | -974.2 [-4147, 9.4] | 36.2 [-3699, 817.1] | 0.21 |
| MCP-1 (pg/ml) | -531.7 [-1674, 221] | -473.1 [-2377, 1618] | 0.55 |
| IL-6 (pg/ml) | -212.8 [-1087, 134.6] | -423.5 [-1517, 209.4] | 0.54 |
| IL-1β (pg/ml) | -57.04 [-825.6, 481.7] | -51.2 [-684, 309.5] | 0.92 |
| ENA-78 (pg/ml) | -74.1 [-3717, 2294] | -292.2 [-2544, 864.8] | 0.67 |
| Data are median [IQR]. IP-10: Interferon gamma-induced protein 10; MCP-1: Monocyte chemoattractant protein 1; IL: interleukin; ENA-78: Epithelial neutrophil activating peptide 78. P value from Mann-Whitney U test used for between-group comparisons. | | | |

| **Supplementary Table E7*. Post-hoc analysis of change in lung function and quality of life based upon EBV suppression at week 8 irrespective of treatment allocation*** | | | |
| --- | --- | --- | --- |
|  | **EBV suppression**  ***n=44** | **EBV persistence**  ***n=23** | **Difference**  **(95% CI)** |
| ***Lung Function variables change from baseline to week 8*** | | | |
| FEV_1_ (L) | 0.026 (0.21) | -0.054 (0.20) | -0.080 (-0.19, 0.026) |
| FEV_1_ % predicted | 0.82 (8.06) | -2.30 (7.15) | -3.12 (-7.11, 0.87) |
| FVC (L) | -0.0077 (0.41) | -0.069 (0.30) | -0.061 (-0.25, 0.13) |
| FEV_1_/FVC Ratio (%) | 0.88 (3.91) | -0.62 (4.23) | -1.50 (-3.57, 0.57) |
| Mid Expiratory Flow (MMEF 25-75% (L/s)) | -0.025 (0.46) | -0.050 (0.14) | -0.024 (-0.22, 0.17) |
| Peak Expiratory Flow (L/s)) | 0.076 (0.76) | -0.030 (0.63) | -0.11 (-0.47, 0.26) |
| Transfer Factor (TLCO percentage predicted) (%)) | n=36  1.11 (6.48) | n=19  -1.00 (6.90) | -2.11 (-5.88, 1.66) |
| ***CAT variables change from baseline to week 8*** | | | |
| Cough | -0.65 (1.35) | -0.29 (1.61) | 0.37 (-0.32, 1.05) |
| Phlegm/ mucus | -0.20 (1.83) | -0.54 (1.43) | -0.33 (-1.13, 0.47) |
| Chest Tightness | -0.22 (1.45) | -0.79 (1.57) | -0.56 (-1.27, 0.14) |
| Breathlessness | -0.45 (1.17) | -0.46 (1.04) | -0.015 (-0.55, 0.52) |
| Limited Activities at Home | -0.020 (1.44) | -0.11 (1.03) | -0.13 (-0.74, 0.49) |
| Confidence in Leaving Home | 0.14 (1.51) | -0.21 (1.20) | -0.36 (-1.02, 0.31) |
| Sleeps Soundly | -0.29 (1.63) | -0.93 (1.33) | -0.64 (-1.37, 0.080) |
| Energy | -0.49 (1.46) | -0.61 (1.10) | -0.12 (-0.75, 0.52) |
| Total CAT score | -2.14 (6.71) | -3.93 (6.34) | -1.79 (-4.89, 1.32) |
| ***EQ5D Subscales (a score of 2 or above) change from baseline to week 8*** | | | |
| Mobility | -0.061 (0.32) | -0.036 (0.33) | 0.026 (-0.13, 0.18) |
| Self-care | 0.082 (0.34) | -0.14 (0.45) | -0.22 (-0.41, -0.043) |
| Usual activities | -0.061 (0.38) | -0.11 (0.42) | -0.046 (-0.23, 0.14) |
| Pain/discomfort | -0.12 (0.44) | -0.21 (0.42) | -0.092 (-0.30, 0.11) |
| Anxiety/Depression | 0.020 (0.43) | -0.11 (0.31) | -0.13 (-0.31, 0.059) |
| Independent samples t-test used to determine the mean difference (95% CI). * Patients with low baseline sputum EBV viral load (<1000 copies per mL) were excluded from the analysis. | | | |

| **Supplementary Table E8*.* Post-hoc analysis of change in lung function from baseline to week 8 based upon EBV suppression excluding individuals who experienced COPD exacerbation** | | | |
| --- | --- | --- | --- |
|  | **EBV suppression**  **n=34** | **EBV persistence**  **n=17** | **Difference**  **(95% CI)** |
| ***Lung Function variables change from baseline to week 8*** | | | |
| FEV_1_ (L) | 0.044 (0.21) | -0.034 (0.19) | -0.078 (-0.20, 0.042) |
| FEV_1_ predicted (%) | 1.53 (7.68) | -1.65 (6.79) | -3.18 (-7.59, 1.24) |
| FVC (L) | 0.029 (0.37) | -0.04 (0.33) | -0.069 (-0.28, 0.15) |
| FEV1/FVC Ratio (%) | 0.74 (3.18) | -0.92 (3.63) | -1.66 (-3.65, 0.33) |
| Mid Expiratory Flow (MMEF 25-75% (L/s)) | -0.046 (0.52) | -0.027 (0.11) | 0.019 (-0.24, 0.28) |
| Peak Expiratory Flow(L/s) | 0.044 (0.74) | 0.084 (0.62) | 0.040 (-0.38, 0.46) |
| Transfer Factor (TLCO) percentage predicted (%) | *n*=28  1.11 (5.98) | *n*=15  -0.93 (7.60) | -2.04 (-6.29, 2.21) |
| ***CAT variables change from baseline to week 8*** | | | |
| Cough | -0.66 (1.42) | -0.32 (1.46) | 0.34 (-0.43, 1.11) |
| Phlegm/ mucus | -0.32 (1.77) | -0.59 (1.59) | -0.28 (-1.19, 0.64) |
| Chest Tightness | -0.24 (1.44) | -1.05 (1.46) | -0.81 (-1.59, -0.031) |
| Breathlessness | -0.42 (1.24) | -0.68 (1.04) | -0.26 (-0.89, 0.37) |
| Limited Activities at Home | -0.026 (1.30) | -0.091 (0.97) | -0.065 (-0.71, 0.58) |
| Confidence in Leaving Home | -0.053 (1.54) | -0.23 (1.23) | -0.17 (-0.95, 0.60) |
| Sleeps Soundly | -0.26 (1.78) | -1.14 (1.39) | -0.87 (-1.76, 0.012) |
| Energy | -0.26 (1.41) | -0.73 (0.88) | -0.46 (-1.13, 0.20) |
| Total CAT score | -2.24 (6.98) | -4.82 (6.06) | -2.58 (-6.15, 0.99) |
| ***EQ5D Subscales (a score of 2 or above) change from baseline to week 8*** | | | |
| Mobility | -0.053 (0.32) | -0.045 (0.38) | 0.0072 (-0.18, 0.19) |
| Self-care | 0.11 (0.39) | -0.045 (0.38) | -0.15 (-0.36, 0.055) |
| Usual activities | -0.079 (0.43) | -0.045 (0.38) | 0.033 (-0.19, 0.25) |
| Pain/discomfort | -0.13 (0.47) | -0.27 (0.46) | -0.14 (-0.39, 0.11) |
| Anxiety/Depression | 0.00 (0.46) | -0.14 (0.35) | -0.14 (-0.37, 0.093) |
